# Elastomeric respirators are safer and more sustainable alternatives to disposable N95 masks during the coronavirus outbreak

**DOI:** 10.1186/s12245-020-00296-8

**Published:** 2020-07-20

**Authors:** James Chiang, Andrew Hanna, David Lebowitz, Latha Ganti

**Affiliations:** 1 HCA Healthcare University of Central Florida Emergency Medicine Residency Program of Greater Orlando, Kissimmee, Florida USA; 2 Envision Physician Services, Clearwater, Florida USA

## Abstract

**Background:**

In this paper, the authors review the safety and practicality of elastomeric respirators for protecting themselves and others from the novel coronavirus or COVID-19. They also describe the safe donning and doffing procedures for this protective gear.

**Main text:**

Due to the shortage of personal protective equipment (PPE), the CDC has recommended ways to conserve disposable N95 masks, including re-use and extended use, and reserving N95 masks for aerosol-generating procedures. However, these were never made to be re-used.

Although the modes of transmission of COVID-19 are not fully understood, based on what we know about severe acute respiratory syndrome (SARS) and Middle East respiratory syndrome (MERS), droplets and aerosolized droplets contribute to the spread of this virus. More evidence from Wuhan, China, has demonstrated that COVID-19 viral particles are aerosolized and found in higher concentrations in rooms where PPE is being removed. Thus, it is best for all healthcare providers to have full aerosol protection.

**Conclusion:**

Given the shortage of PPE for aerosols, it is logical to utilize reusable elastomeric respirators with filter efficiency of 95% or higher. A single elastomeric respirator may replace hundreds to thousands of new disposable N95 masks.

## Background

Since the coronavirus (2019-nCoV) or coronavirus disease (COVID-19) outbreak, shortage of personal protective equipment (PPE) has become a significant issue in numerous countries worldwide [1], with an estimate of 89 million masks required monthly due to increased demand within and outside healthcare settings [2]. To combat these shortages, the Center of Disease Control (CDC) has put forth recommendations to conserve PPE in the USA. Some of these recommendations include using N95 masks for extended durations, using one mask for several different patients, reserving disposable N95 masks for aerosol generating procedures, and using reusable elastomeric respirators with appropriate filters [3]. Re-using PPE has placed significant strain on health care professionals as many have resorted to reusing the same N95 mask for several days and keeping it in a paper or resealable plastic bag [4, 5]. Disposable N-95 masks were never made to be re-used. This practice raises significant safety concerns as lack of PPE and negative pressure isolation rooms have contributed to more than 5000 confirmed cases of COVID-19 in health care professionals in Italy, and almost forty health care workers have died from coronavirus infections as of March 26, 2020 [6]. In the USA, similar cases are occurring as health care professionals are increasingly diagnosed with COVID-19 and are dying from coronavirus [7]. Given the persistent shortage of disposable N95 masks, the authors review the safety and practicality of elastomeric respirators as an alternative for protection against the coronavirus COVID-19.

## Main text

### COVID-19 transmission

The mode of transmission of COVID-19 is not fully understood, but based on extrapolating research on influenza and similar respiratory infections also caused by other coronaviruses such as SARS (severe acute respiratory syndrome) and MERS (Middle East Respiratory Syndrome), the World Health Organization (WHO) and CDC postulate that COVID-19 is more likely to be transmitted by close contact and respiratory droplets, but these respiratory droplets may be inhaled [8, 9], resulting in airborne transmission. According to the Chinese CDC, aerosolized respiratory droplets are very likely inhaled when in close contact with patients, and thus, virtually, the entire population is susceptible to COVID-19 infections [10]. A study done in Wuhan where COVID-19 had its initial outbreak found that aerosolized viral particles were detectable in high concentrations in rooms where providers were doffing PPE, and the majority of these particles were 0.25 to 0.5 μm. Another study has found that aerosolized viral particles of COVID-19 are detectable in the air 3 h after aerosolization [11]. Based on this evidence, proper protective equipment against aerosols and the safe removal of PPE are crucial for prevention of coronavirus infection in the healthcare setting. Reusing disposable N95 masks potentially exposes patients and workers to considerable infection risk.

### Elastomeric respirators

Elastomeric respirators have more commonly been used in industrial and mining settings, but can be considered for use in the health care setting during times of increased demand such as during infectious disease outbreaks [12]. These respirators’ gas and vapor cartridges have a finite duration of use for protection against various toxic vapors, organic or inorganic gases, and chemical aerosols based on the chemical’s exposure limit and concentration. Once the sorbents are saturated, these toxic gases will break through, and thus, cartridges are regularly renewed [13]. For particulates such as dust, aerosols, mold, and bacteria, electrostatic particulate filters are used instead, and the more the filter is filled with contaminant, the more effective these electrostatic forces are. Hence, particulate filters are more effective with use over time given proper conditions, although once they become too difficult to breathe through, they must be replaced [14]. In this regard, respirator masks with particulate filters can be used for an extremely long duration in a hospital setting, at least 1 year, so long as the filter is not damaged or soiled [15]. Evidence shows that coronavirus remains detectable and viable on various surfaces up to 3 days [11]. It can then be extrapolated that once these viral particles are trapped in the electrostatic filters, they will slowly die over several days, negating the need to change viral filters frequently. Given the current short supply of disposable N95 masks, this advantage can potentially allow one respirator mask to replace using hundreds to potentially thousands of new disposable N95 masks.

Elastomeric respirators also differ from disposable N95 masks in that they have a separate exhale vent, and exhaled air does not travel through the contaminated filter and re-aerosolize trapped viral particles. This is in contrast to disposable N95 masks, which when re-used may carry an increased risk of transferring viral particles from one patient to another. However, this same advantage of elastomeric respirators can be a disadvantage if the wearer has an active respiratory infection, as he or she could spread their own infection from patient to patient. Thus, the health care provider must be vigilant for any signs or symptoms of COVID-19 in themselves.

### Better fit and seal

National Institute for Occupational Safety and Health (NIOSH)-approved respirator masks have three non-powered particulate filter efficiency classes: 95, 99, and 100, for 95%, 99%, and 99.97% filtration of particulates down to 0.3 μm, respectively. There are three levels of oil-particle resistance, N for no resistance, R for some oil resistance, and P for being completely oil proof [16]. For most aerosols in healthcare settings, filter efficiency of at least 95% with no oil resistance is generally accepted. Each user needs to be fit-tested for these respirator masks to be effective [17]. However, several studies show that healthcare professionals that do not frequently use N95 masks or wear masks for an extended duration tend to have inadequate seal. Nearly half of healthcare professionals who repeat a fit test 3 months after passing a fit test end up failing the second fit test [18].

Another study finds that 4.0-μm particulates were detectable inside respirator masks using portable aerosol spectrometers after only 10 min of body movements during nursing procedures [19]. This is an indication that the seal is easily lost during extended use. Compared to disposable respiratory masks of the same filter efficiency, elastomeric respirators have been found to have 60% higher filtration performance and better seal [20–22]. Although not as effective as powered air-purifying respirators (PAPRs), elastomeric respirators are still superior and preferred options over disposable respirators, especially given the severe shortage and increasing cases of healthcare professional infected with coronavirus.

Currently, the CDC recommends that health care providers that will be within 3 ft of a suspected or confirmed COVID-19 patient without a mask, or if providers are in the room for an aerosolizing procedure, the provider may voluntarily utilize higher level of protection than N95 masks, such as elastomeric respirators or PAPR. The American Academy of Emergency Physicians and American Association of Nurse Anesthetists have made official statements that support healthcare providers using self-supplied NIOSH-approved PPE to feel safe or if it is inadequately provided [23, 24].

### Proper disinfection and usage of elastomeric respirators

Similar to use of disposable N95 masks, caution must be taken regarding the use and reuse of elastomeric respirators so as to decrease contamination of the inside of the respirator and thus increasing the risk of infecting healthcare workers. Use falls under two primary categories with regard to elastomeric respirators: reuse and extended use [25].

**Fig. 1:**
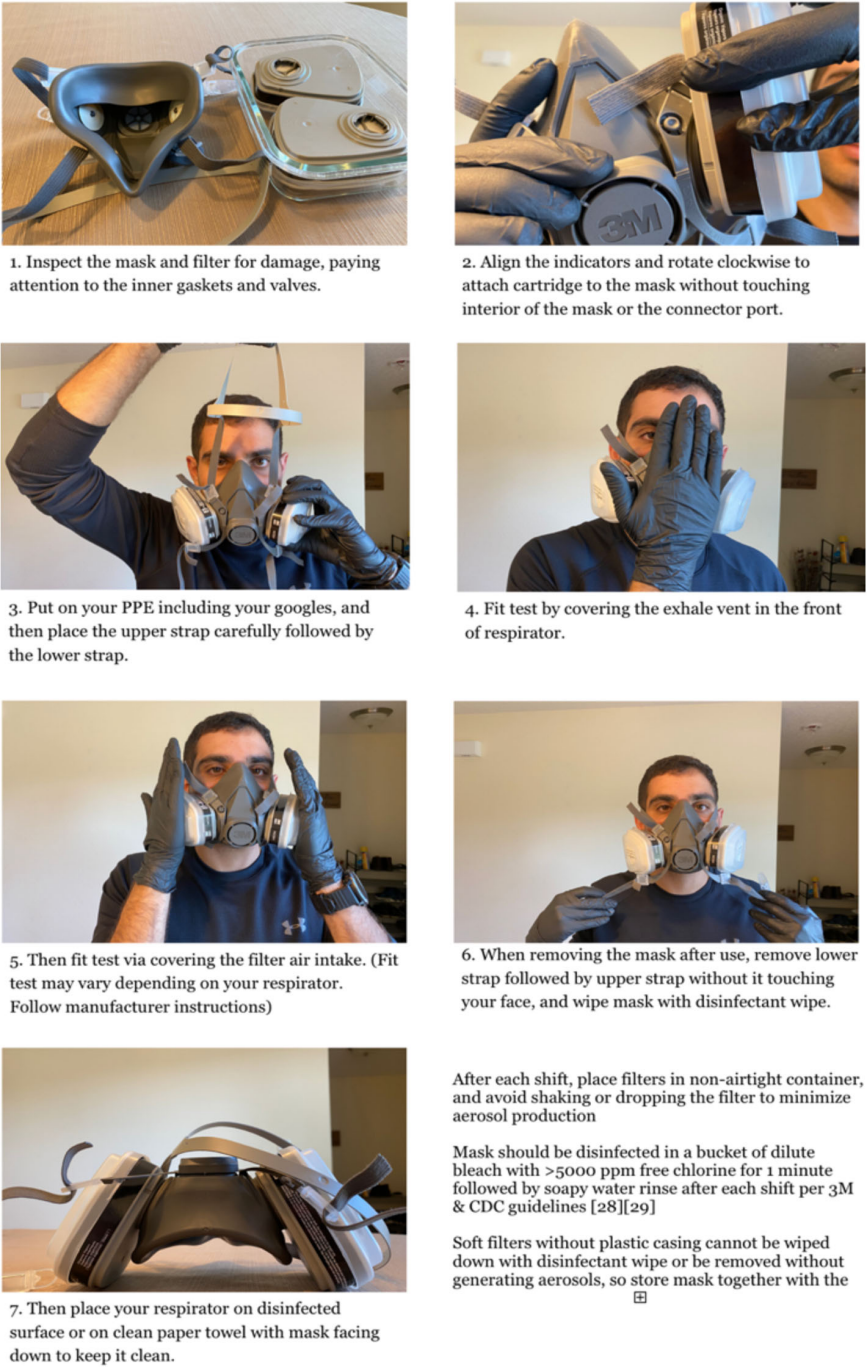
Safe reuse of the elastomeric respirators

Reuse refers to the donning and doffing of a respirator multiple times throughout the duration of a clinical period. This is frequently seen in settings where different patients represent different infection transmission risk, and thus, the respirator is donned for use during aerosolizing procedures and doffed after the encounter. The greatest pitfall with reuse, as with disposable N95 masks, is the risk of contamination during the doffing and re-donning procedure as repeated multiple times throughout the duration of the clinical period. In addition, appropriate storage of the masks is necessary so as to prevent contamination of the inner portion of the respirator when it is exposed. When comparing reuse of single-use N95 masks versus elastomeric respirators, there is a clear benefit of the latter as the material and efficacy of the filter will not degrade from repeated donning and doffing. Utilizing a standard operating procedure, safe reuse of the elastomeric respirators can be performed (Fig. 1). Moreover, given the ability to better effectively adjust fit, reuse of an elastomeric respirator ensures better seal on repeat use compared to N95 masks which often require manipulation of the contaminated front of the mask to reobtain proper seal.

Extended use of a respirator refers to the donning of a mask or elastomeric respirator at the start of a clinical period and kept on the provider for all patient encounters, only to be doffed after patient interaction has ceased. This is a preferred method of use with both N95 and elastomeric respirators as it eliminates the risk of contamination with reuse. The disadvantage to extended use is provider discomfort as well as the risk of transmission of pathogens between patients from the respirator [26]. With regard to N95, extended use should include wearing a disposable surgical mask or face shield over the respirator that can be removed after each patient encounter to prevent soiling of N95. The benefit to use of elastomeric respirators is that, while difficult to definitively test, the CDC recommends no more than 8 h of continued or intermittent use of N95 masks, purporting an obvious disadvantage if one were to require more than 8 h of continuous use [25]. Moreover, given the material of the elastomeric respirators the external surface of the mask can be wiped down while still on, the providers face between encounters or during encounters where there was droplet exposure. An often-noted disadvantage of frequent reuse or extended use of elastomeric respirators is skin break down over the bridge of the nose and side of the face. Early skin break down anecdotally can be mitigated by use of adhesive strips or barrier protection such as a bandage or tape prior to donning of the respirator.

In times of PPE shortage as with the H1N1 influenza outbreak, methods for disinfection of disposable N95 masks have been suggested including ultraviolet germicidal irradiation and prolonged heat exposure [27, 28]. Effective sterilization often requires equipment not readily accessible to healthcare providers. Elastomeric respirators, however, as recommended by the manufacturer, can be easily disinfected using a solution containing free chlorine at 5000 parts per million. Standard operating procedure for disinfection of elastomeric respirators has previously been published, and cleaning solution can be made with materials easily found in most healthcare facilities [29, 30], which makes this mask even more versatile in pandemic times. It is recommended that the cleaning and disinfection process of the respirator be centralized and performed by trained personnel [15].

## Conclusion

Elastomeric face masks have several advantages over reusing disposable N-95 masks. They provide safe reusable protection, can be easily cleaned, and have lower risk of transmitting infection between patients. While not originally designed for hospital use, they provide an excellent solution to the shortage of disposable N-95 masks during this COVID-19 pandemic.

## Data Availability

N/A

## Abbreviations

PPE: Personal protective equipment
COVID-19: 2019 novel coronavirus
CDC: Centers for Disease Control
WHO: World Health Organization
NIOSH: National Institute for Occupational Safety and Health
PAPR: Powered air-purifying respirators
